# NGF Causes TrkA to Specifically Attract Microtubules to Lipid Rafts

**DOI:** 10.1371/journal.pone.0035163

**Published:** 2012-04-04

**Authors:** Shona Pryor, Gretchen McCaffrey, Lindsay R. Young, Mark L. Grimes

**Affiliations:** 1 Division of Biological Sciences, University of Montana, Missoula, Montana, United States of America; 2 Center for Structural and Functional Neuroscience, University of Montana, Missoula, Montana, United States of America; 3 Institute of Molecular Biosciences, Massey University, Palmerston North, New Zealand; Aix Marseille University, France

## Abstract

Membrane protein sorting is mediated by interactions between proteins and lipids. One mechanism that contributes to sorting involves patches of lipids, termed lipid rafts, which are different from their surroundings in lipid and protein composition. Although the nerve growth factor (NGF) receptors, TrkA and p75^NTR^ collaborate with each other at the plasma membrane to bind NGF, these two receptors are endocytosed separately and activate different cellular responses. We hypothesized that receptor localization in membrane rafts may play a role in endocytic sorting. TrkA and p75^NTR^ both reside in detergent-resistant membranes (DRMs), yet they responded differently to a variety of conditions. The ganglioside, GM1, caused increased association of NGF, TrkA, and microtubules with DRMs, but a decrease in p75^NTR^. When microtubules were induced to polymerize and attach to DRMs by *in vitro* reactions, TrkA, but not p75^NTR^, was bound to microtubules in DRMs and in a detergent-resistant endosomal fraction. NGF enhanced the interaction between TrkA and microtubules in DRMs, yet tyrosine phosphorylated TrkA was entirely absent in DRMs under conditions where activated TrkA was detected in detergent-sensitive membranes and endosomes. These data indicate that TrkA and p75^NTR^ partition into membrane rafts by different mechanisms, and that the fraction of TrkA that associates with DRMs is internalized but does not directly form signaling endosomes. Rather, by attracting microtubules to lipid rafts, TrkA may mediate other processes such as axon guidance.

## Introduction

Cells profoundly change behavior according to instructions provided by molecular signals. Neurons choose life over programmed cell death in response to neurotrophin signaling, and extend processes that grow towards neurotrophin-secreting cells. Neurotrophin signaling is mediated by receptor tyrosine kinases of the Trk family, TrkA, B, and C, which, respectively, interact specifically with nerve growth factor (NGF), brain-derived neurotrophic factor (BDNF), and neurotrophin-3 (NT3). Trk signaling differs from other receptor tyrosine kinases because of the involvement of a co-receptor, the pan-neurotrophin receptor, p75^NTR^. TrkA and p75^NTR^ collaborate at the plasma membrane to bind NGF [1], [2], [2]–[4], yet appear to have an antagonistic relationship in other ways. TrkA and p75^NTR^ are endocytosed separately after binding NGF [5]–[7]. p75^NTR^ when activated by itself causes apoptosis, but in the presence of Trk signaling, neurons are protected from programmed cell death [8], [9]. NGF influences microtubule dynamics at axon tips to cause axon growth in Trk-expressing cells [10]. In contrast, when Trk is not present, p75^NTR^ together with its other co-receptors, the Nogo-66 receptor (NgR), and Lingo-1 mediates growth cone repulsion [11], [12]. Thus, the relationship between TrkA and p75^NTR^ can be characterized as a duel, where the two partners meet briefly, then go their separate ways, pursuing different agendas. How do they go their separate ways after their first meeting? The molecular interactions that separate the two receptors at the plasma membrane are not known.

The interaction of proteins with clusters of different kinds of lipids in membranes plays a role in signal transduction, membrane traffic sorting, and axon guidance [13]–[15]. For instance, GPI-anchored proteins and Src-family kinases are clustered in detergent-resistant sphingolipid-cholesterol lipid rafts [16]. Similarly, several receptor tyrosine kinases and G-protein coupled receptors move into lipid rafts upon activation, along with their effectors, and, interestingly, some receptors move out of lipid rafts when they are activated [17]. This implies that dynamic association of receptors with lipid rafts may play a role in sorting at the plasma membrane. The ganglioside, GM1 and other lipid raft markers are excluded from clathrin-coated pits, which contain the transferrin receptor (TfR) and other non-raft proteins [18]. We hypothesize that lipid rafts may play a role in sorting p75^NTR^ and TrkA into different endocytosis pathways.

Receptors are endocytosed by two or more distinct pathways. In general, receptors may be internalized by clathrin-mediated endocytosis (CME), or a pathway that involves sphingolipid-cholesterol lipid rafts, termed raft/caveolar endocytosis (RCE) [19]–[22]. The CME vs. RCE endocytosis choice has not been directly described for Trk receptors. Trk receptors are internalized by CME [23]–[27] and by a clathrin-independent mechanism that involves the EH-domain containing protein, Pincher [28]–[32]. p75^NTR^ is internalized in sympathetic neurons by both CME and a mechanism that involves lipid rafts [25], [33].

Here, we asked whether the association of TrkA and p75^NTR^ with detergent-insoluble membranes (DRMs) is affected by NGF and *in vitro* reactions that have been shown to cause microtubules to polymerize [34]. DRMs are defined as the fraction of the detergent-insoluble material that float on iodixanol (Optiprep™) equilibrium gradients. This method is similar to that used by others to characterize components of sphingolipid-cholesterol lipid rafts, but offers higher resolution of raft components of different densities and quantitative comparison of relative amounts of components that are found in detergent-resistant membranes. We found that NGF and microtubules had profoundly different effects on the association of TrkA and p75^NTR^ with DRMs. The data suggest that the portion of TrkA which associates with microtubules and lipid rafts has a distinct function separate from formation of signaling endosomes.

## Results

### NGF and its Receptors in Detergent-resistant Membranes (DRMs)

In cell fractionation studies in which ^125^I-NGF is bound to PC12 cells in the cold, and the cells are washed and warmed to allow internalization of NGF-bound receptors [7], [35], [36], NGF caused rapid internalization of TrkA into endosomes that could be recovered in organelles that emerged when cells were mechanically permeabilized by a single passage through a tight passage created by a ball whose diameter is very close to that of a surrounding cylinder (Balch homogenizer [35], [36]). After 10 min, about 40% of the TrkA is internalized, compared to a background endocytosis of about 5% without NGF. Under these conditions, at least 30% of NGF was reproducibly associated with the detergent-insoluble pellet after extraction with 1% non-ionic detergent (Triton X-100, NP-40 or IGEPAL; see Table 1). In contrast, only 1–4% of ^125^I-transferrin is associated with the detergent-insoluble pellet under identical experimental conditions (Table 1). The significant difference between the amount of NGF vs. transferrin associated with the detergent-insoluble pellet leads to the hypothesis that NGF receptors are recruited into DRMs that would float when the pellet was resuspended layered under an iodixanol equilibrium gradient.

**Table 1:** Amount of Radioactive Ligands Associated With Cell Fractions.

Cell Fraction	^125^I-Ligand			
	Transferrin		NGF	
	*% total*	*SEM*	*% total*	*SEM*
1000 × g pellet				
NP40 soluble	67.2	3.2	56.6	4.0
NP40 insoluble	4.3	2.0	30.1	4.3
1000 × g supernatant	30.4	1.8	14.6	0.5

Cells were bound to radiolabelled ligand, washed, and subjected to internalization 10 min at 37°C. Mechanical permeabilization, fractionation, and detergent extraction was performed exactly as described [35], [36].

**Figure 1:**
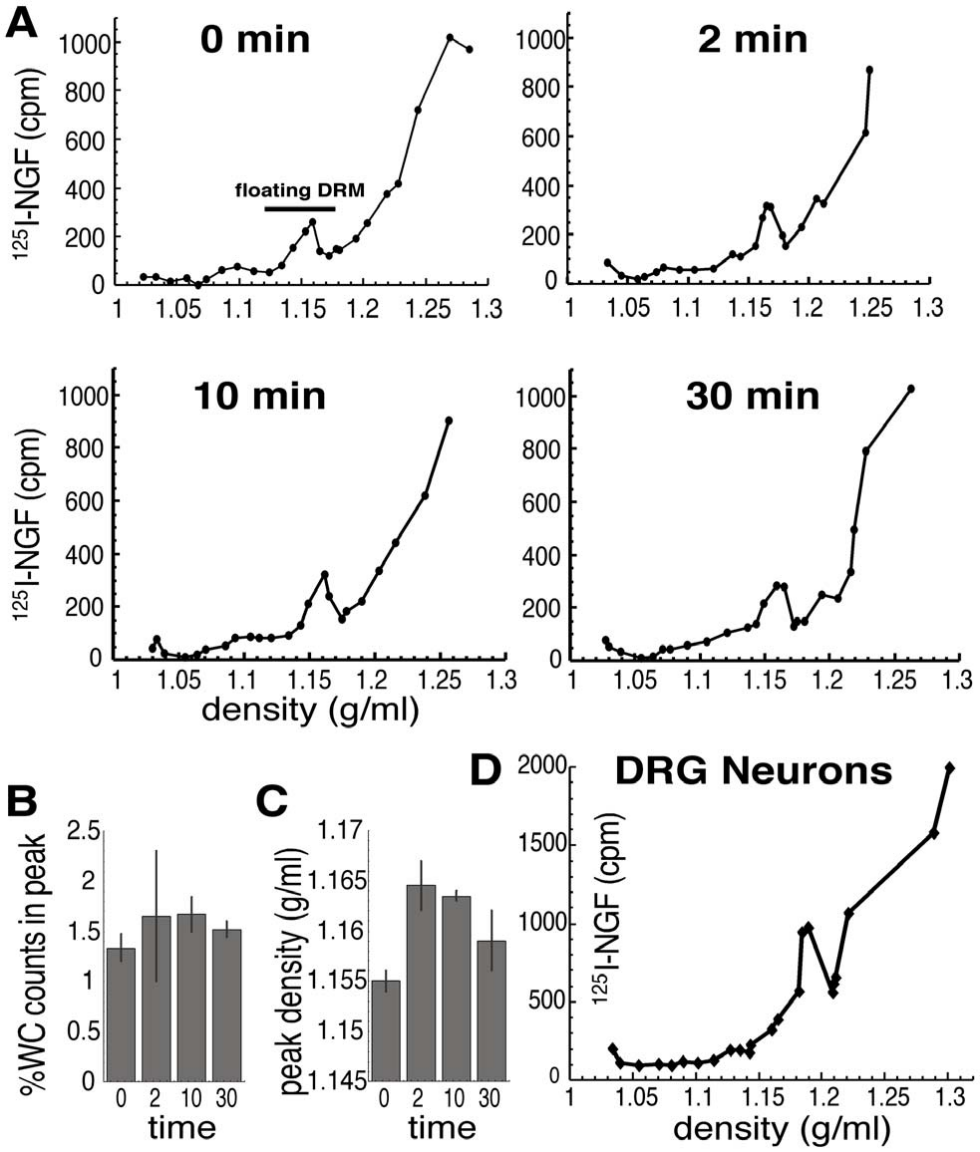
NGF associates with lipid rafts before and after initiation of membrane traffic and signal transduction. ^125^I-NGF was bound to PC12 cells in the cold, the cells were washed and warmed for the indicated periods of time. 0 min represents cells bound to NGF but not warmed. Flotation equilibrium iodixanol gradients were performed using sonication to resuspend the detergent-resistant fraction. A) Plots of DRM gradients after pulse-stimulation with ^125^I-NGF for 0, 2, 10, and 30 min. Refractive index measurements were taken on each fraction and converted into density using a formula derived empirically (see Methods); density is plotted on the x-axis. There also was non-floating NGF in the detergent-resistant pellet, whose distribution in fractions of higher density is consistent with diffusion in a bottom-loaded sample. B, C) Amount of NGF and density of floating DRMs. The amounts of ^125^I-NGF in the floating DRM peak containing ^125^I-NGF were quantified and compared to amounts in detergent-soluble membranes and other fractions (B, %WC=percent of whole cell). Amounts in the floating DRM peak are plotted as the percent in the whole cell. A transient increase in the density of the floating ^125^I-NGF DRM peak was noted after 2 and 10 min (C). A higher density suggests a higher protein:lipid ratio. Error bars are SEM. D) DRM fraction from rat dorsal root ganglia neurons bound to ^125^I-NGF and warmed for 10 min as above. The floating peak had a slightly higher density (1.18 g/ml) than that in PC12 cells (1.16 g/ml).

We used a similar pulse-stimulation protocol to investigate the association of NGF and its receptors, TrkA and p75^NTR^ with DRMs: cells were bound to ^125^I-NGF in the cold, then washed and warmed for defined periods. Cells were lysed in non-ionic detergent and the insoluble material was subjected to equilibrium flotation iodixanol gradients (Figure 1). The peak at ρ=1.155–1.165 g/ml is defined as DRMs, which separated from higher density non-floating material (Figure 1A). NGF was present in DRMs on the plasma membrane before warming (Figure 1A, 0 min) and persisted for 30 min. There was little change in the amount associated with the floating peak over time (Figure 1B), although the density of the floating peak increased transiently (Figure 1C). Rat dorsal root ganglia neurons displayed a similar floating DRM peak containing NGF, though the density of this peak was slightly higher than that derived from PC12 cells (Figure 1D). These data suggest that PC12 cells are a valid model for neurons for the study of the DRM fraction containing NGF receptors.

**Figure 2:**
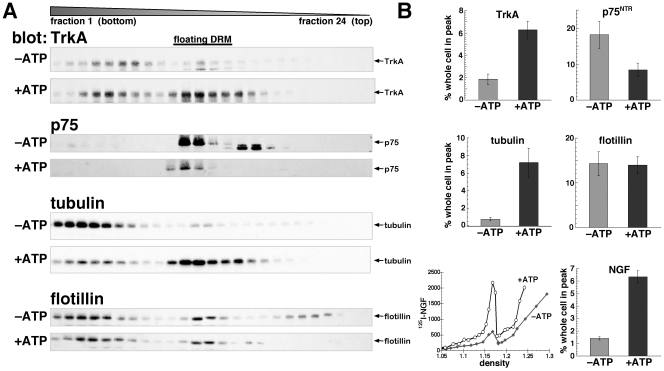
In vitro reactions cause microtubules to associate with lipid rafts, attracting NGF and TrkA but excluding p75^NTR^. Cells were bound to NGF and sonicated DRMs fractionated on flotation equilibrium gradients as in Figure 1 (0 min internalization). A) Western blots with anti-TrkA, -p75^NTR^, -tubulin, and -flotillin (indicated) from cells fractionated before (–ATP) and after (+ATP) in vitro reactions with ATP. Gradients were collected from the bottom, so lower numbered fractions have higher density. The floating DRM peak is in the middle of the gradients. B) The plot in the lower left is ^125^I-NGF in DRMs from cells before (closed diamonds) or after (open circles) in vitro reactions with ATP. The amounts of ^125^I-NGF, and proteins shown in A in the floating DRM peak coincident with ^125^I-NGF, were quantified and compared to amounts in detergent-soluble membranes and other fractions. Amounts in the floating DRM peak are plotted as the percent in the whole cell under these conditions (–ATP, +ATP). Error bars are SEM. In vitro reactions with ATP caused a significant increase of TrkA (p<0.01) and NGF and tubulin (p<0.001), and a decrease in p75^NTR^ (p<0.05) in floating DRMs after 0 min internalization.

To test the hypothesis that lipid rafts play a role in sorting TrkA and p75^NTR^ into different endocytic pathways, we focused on the time points of 0 and 10 min. At these times, TrkA and p75^NTR^ associated with floating DRMs in a peak at the same density as ^125^I-NGF (Figure 2; see below).

### Microtubules in Rafts

Previously, we showed that tubulin could be detected in the detergent-resistant pellet from PC12 cells [34]. Since tubulin can be palmitoylated and the palmitoyl group when attached to proteins often confers association with DRMs [37], [38], we asked whether tubulin could be detected in floating DRMs (Figure 2A). In previous work, *in vitro* reactions with ATP enhanced tubulin polymerization leading to increased amounts of microtubules in the detergent-resistant pellet [34]. These data show that *in vitro* reactions with ATP can be used to manipulate microtubule polymerization. *In vitro* reactions increased by 9-fold the amount of tubulin in the floating DRM peak (Figure 2). Under these conditions, NGF and TrkA both increased 4–5 fold (Figure 2). In contrast, p75^NTR^ was reduced by about half in the floating peak after *in vitro* reactions (Figure 2). Flotillin was not affected, indicating that *in vitro* reactions do not artifactually produce a general aggregation of membranes (Figure 2). Thus, the amount of NGF and TrkA receptors in floating DRM specifically correlates with the presence of microtubules.

### GM1 Addition Increased NGF, TrkA, and Microtubules in DRMs

**Figure 3:**
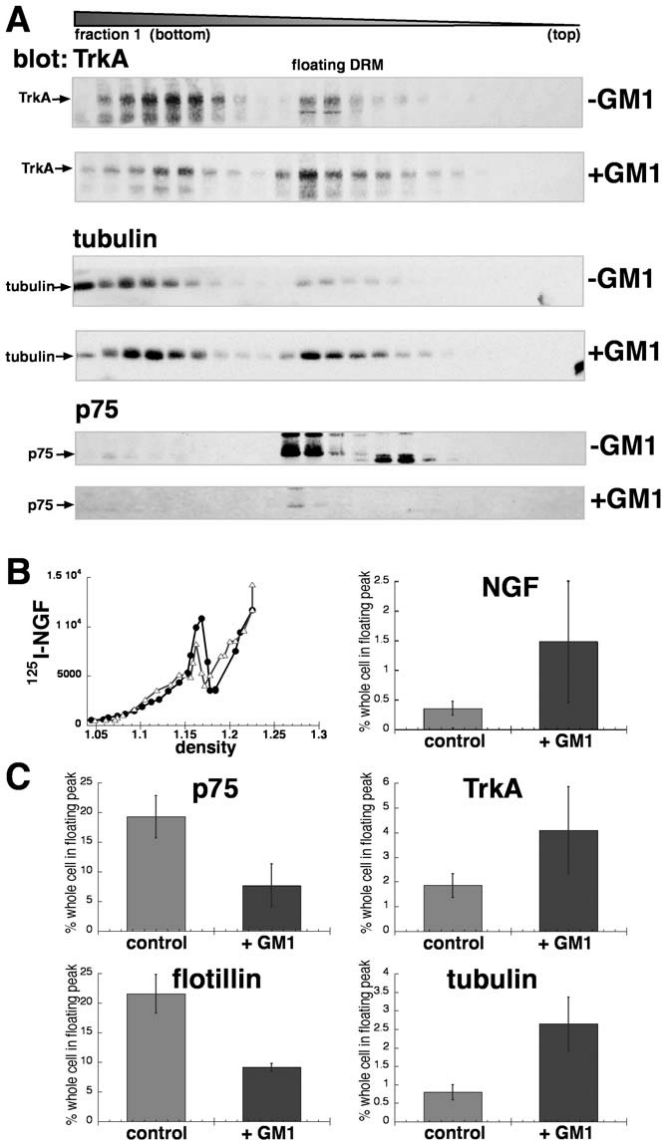
The ganglioside, GM1 affects the partitioning of NGF, its receptors, and microtubules in floating DRMs. GM1 was pre-incubated with cells at 65 µM. Control samples in the same experiment were treated identically except no GM1 was added. Cells were bound to ^125^I-NGF and sonicated DRMs were floated on iodixanol equilibrium gradients as in Figure 1 (0 min). A) Western blots showing that TrkA and tubulin were increased in floating DRMs with GM1 treatment. B) ^125^I-NGF in floating DRMs (left, plotted as in Figure 1) increased with GM1 treatment (filled circles) compared to control (open triangles). The amount of ^125^I-NGF in floating DRMs, plotted as a fraction of that in the whole cell as in Figure 2, increased in GM1-treated cells (p<0.1). C) Data from western blots with anti-TrkA, -p75^NTR^, -flotillin and -tubulin (indicated) were quantified and the amount in the floating DRM peak coincident with ^125^I-NGF are plotted as the percent of the whole cell for control and GM1-treated cells. The amount of p75^NTR^ in the floating DRMs decreased by half (from 19% to 8%) after GM1 treatment (p<0.1). Flotillin was also decreased (p<0.1), while TrkA (p<0.1) and tubulin (p<0.01) were increased by GM1.

We asked if treatments that are known to affect the amount and the activity of TrkA in lipid rafts also affect microtubules in DRMs. The ganglioside, GM1 has been shown to activate Trk receptors and prevent apoptosis in sympathetic neurons and PC12 cells, which is hypothesized to be due to increased TrkA association within lipid rafts, [39]–[43]. Overexpression of the enzyme that produces GM1, however, has also been shown to decrease amounts of specific proteins associated with rafts and suppress TrkA dimerization, which is required for signaling activity [44]. These data suggest that TrkA signal transduction causes its recruitment to lipid rafts. One possibility is that GM1 at very high levels may also dilute rafts or change the properties of the membrane such that signaling is impeded. To determine whether changes in lipid rafts affected the recruitment of TrkA and p75^NTR^, we measured the effect of adding GM1 on the amount of NGF, its receptors, and microtubules in DRMs. GM1 increased NGF and Trk in DRMs more than 2-fold (Figure 3A, B). In contrast, p75^NTR^ and flotillin were affected by GM1 in the opposite way. GM1-treated cells had less than half the amounts of p75^NTR^ and flotillin in floating DRMs compared to those of control (Figure 3C). It is important to note that the fraction of p75^NTR^ and flotillin in DRMs is constitutively high, about 20% without GM1 treatment, compared to TrkA (∼2%). p75^NTR^ and flotillin are known to preferentially associate with lipid rafts in many different cell types, and this property may be related to their similar decrease in DRMs in GM1-treated cells. The data are consistent with high levels of GM1 diluting rafts, which affects proteins that preferentially or constitutively associate with rafts differently than proteins that transiently associate with rafts in response to stimulation.

We also found that the microtubules that associated with floating DRMs increased more than 3-fold after GM1 treatment (Figure 3A and C, tubulin). Thus, GM1’s effects, as with *in vitro* reactions that cause microtubules to polymerize, were to increase microtubules in DRMs, which correlated with increases in NGF and TrkA. In both cases p75^NTR^ behaved in the opposite manner. The data suggest that NGF is mostly bound to TrkA, not p75^NTR^, in floating DRMs, because the changes in the distribution of NGF paralleled that of TrkA.

**Figure 4:**
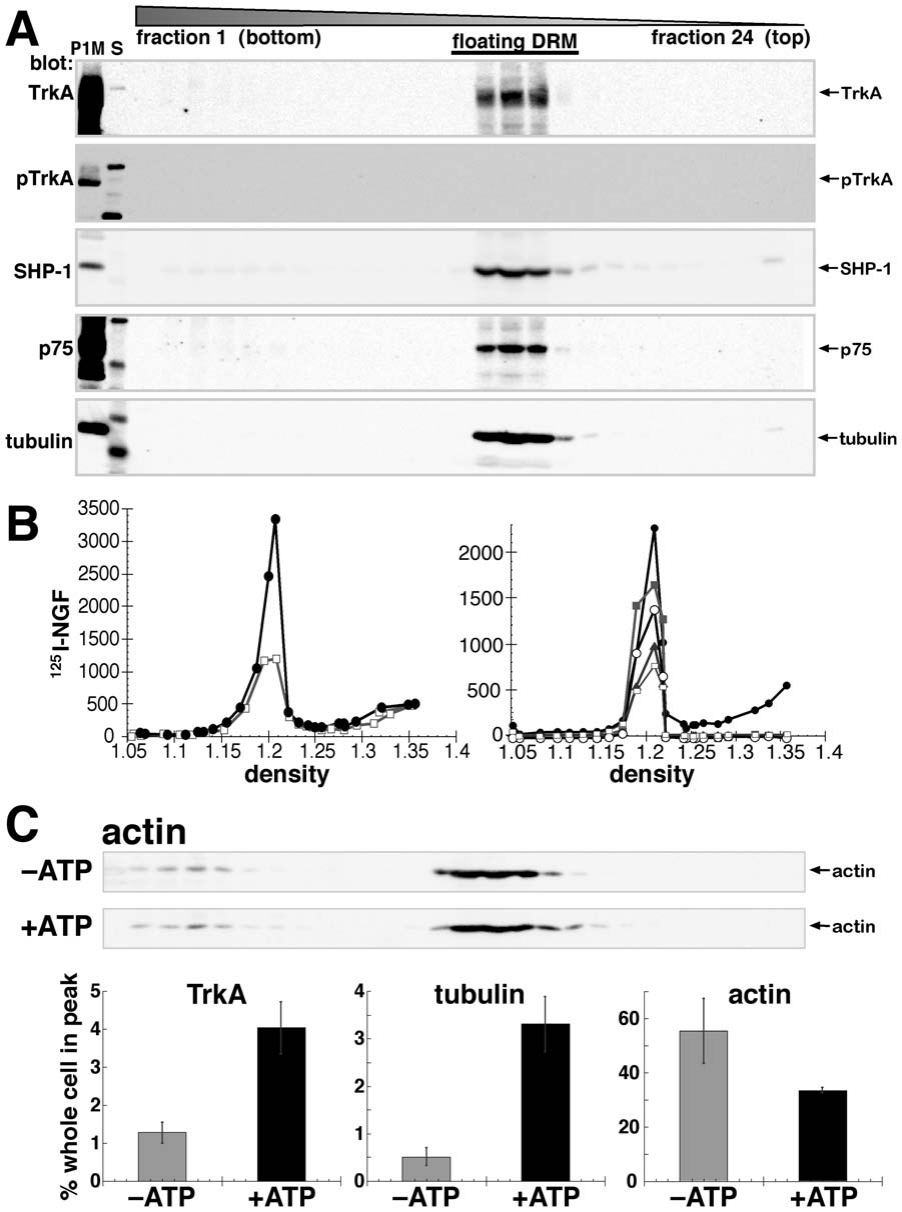
Association of NGF receptors and cytoskeletal elements with DRMs under different experimental conditions. A) Western blots showing TrkA and p75^NTR^ in floating DRMs prepared after 10 min NGF treatment and using nuclease (Benzonase) rather than sonication to break up nucleic acids prior to equilibrium density gradients. Western blots of flotation equilibrium gradients of detergent-resistant fraction were probed with anti-TrkA, -pTrkA, -SHP-1, -p75^NTR^, and -tubulin (indicated). Blots include the detergent-sensitive (P1M) fraction and size standards (S) to the left of DRM gradient fractions. B) Left: ^125^I-NGF in DRM without (open squares) and with (closed circles) in vitro reactions with ATP. Right: Quantification of chemiluminescent signals from western blots is compared to ^125^I-NGF for Benzonase-treated samples as in A. Fraction number is plotted on the x-axis of plot on the left; fraction 1 has the highest density. Signals from western blots were quantified and plotted vs. density together with ^125^I-NGF (closed circles) on the right. The y-axis for TrkA (closed squares), p75^NTR^ (open circles), SHP-1 (open squares), and tubulin (closed triangles) is arbitrary units (chemiluminescent pixel volume). C) Western blots showing actin in floating DRMs prepared as in A, with and without in vitro reactions with ATP (–, +ATP). Data obtained under these conditions for TrkA, tubulin, and actin were quantified and plotted as in Figure 2. After 10 min internalization, in vitro reactions with ATP caused a significant increase of TrkA (p<0.01) and NGF and tubulin (p<0.001) in floating DRMs. A decrease in actin (+ATP) was noted but was not statistically significant.

We used two different methods to break up the insoluble material in the detergent-resistant pellet: sonication and nuclease (Benzonase) treatment (see Materials and Methods). When sonication was used, the density of the floating peak was approximately 1.16 g/ml. Trk was associated with two peaks on these gradients, one of which coincided with the floating NGF peak (Figure 2). The other peak was of higher density (1.23 g/ml) and did not coincide with NGF (Figure 2). p75^NTR^ was also found in the 1.16 g/ml floating peak with NGF and Trk, and little was present in other regions of the gradient (Figure 2). A fraction of tubulin also remained in the non-floating bottom of the gradient under these conditions (Figure 2). These data indicate that a fraction of the microtubules in DRMs were reproducibly resistant to sonication. To further investigate this possibility we determined whether results could be obtained without sonication. We found that Benzonase treatment facilitated handling of the DRM fraction without sonication (Figure 4). Quantitative distribution of receptors into floating DRMs was similar after sonication or Benzonase treatment, but the receptors floated into a peak of slightly higher density (1.20 g/ml) and there was less non-floating material in benzonase-treated samples (Figure 4A, B). Actin filaments were also present in floating DRMs under these conditions (very few were detected after sonication), but there was no change in their association with DRMs after *in vitro* reactions (Figure 4C). Importantly, comparable increases in NGF and microtubules in floating DRMs after *in vitro* reactions were observed (Figure 4B,C).

TrkA was reproducibly dephosphorylated in floating DRMs. Under conditions where phospho-TrkA (pTrkA) was detected in the detergent-sensitive (P1M) fraction, and in endosomes (see below, Figure 7A), TrkA but not pTrkA was present in floating DRMs (Figure 4A). The presence of the tyrosine phosphatase, SHP-1 in floating DRMs (Figure 4A, B) suggests a mechanism by which TrkA is selectively dephosphorylated in this fraction.

**Figure 5:**
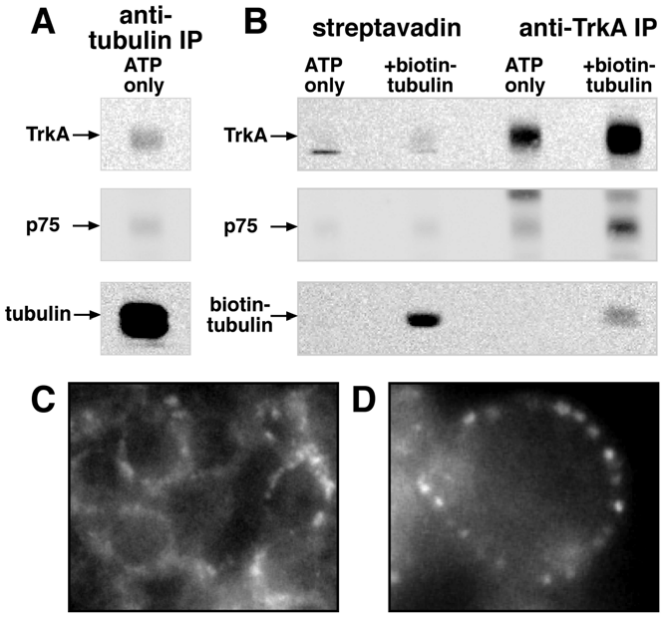
TrkA is bound to microtubules in floating DRMs. Floating DRMs were isolated after 10 **min NGF treatment and in vitro reactions.** A) Microtubules were immunoprecipitated from the floating peak with anti-β-tubulin and western blotted for TrkA (upper panel) and tubulin (lower panel). B) In vitro reactions without (ATP only) or with biotinylated tubulin added during the last 5 min of the reaction (+biotin-tubulin) were performed. The floating DRM peak was immunoprecipitated with streptavidin or anti-TrkA (indicated) and western blotted for anti-TrkA (upper panel) and anti-biotin (lower panel). p75^NTR^ was not reproducibly detected in microtubule immunoprecipitations from DRMs under these conditions. C, D) Images of permeabilized cells after in vitro reactions with biotinylated tubulin added during the last 5 min of the reaction. Texas red streptavidin stained discreet foci at or near the plasma membrane, shown in a group of permeabilized cells in C and an individual cell in D.

The similar increases in NGF, TrkA and microtubules in DRMs in response to GM1 and *in vitro* reactions suggest that TrkA may bind to microtubules in this fraction. Indeed, TrkA was co-precipitated when microtubules were stabilized with taxol and immunoprecipitated from floating DRMs (Figure 5A). If biotinylated tubulin was added during the last 5 min of *in vitro* reactions, it was incorporated into floating DRMs, suggesting that newly polymerized microtubules were associated with this fraction (Figure 5B, biotin-tubulin). Streptavidin agarose beads recovered TrkA from this fraction and biotinylated tubulin was pulled down by TrkA immunoprecipitation (Figure 5B). p75^NTR^ was not reproducibly detected in microtubule immunoprecipitations in these experiments. These data suggest that microtubule polymerization and attachment to DRMs recruits TrkA.

Under these conditions, biotinylated tubulin accumulated in discrete foci at the plasma membrane of permeabilized cells (Figure 5C, D). Long, intact microtubules were not evident in the permeabilized cell preparation, so we cannot exclude the possibility of some kind of alternate assembly of tubulin subunits at these discrete foci on the plasma membrane. Nevertheless, our data above showing that NGF and TrkA are recruited into DRMs along with microtubules, but not p75^NTR^ or flotillin, indicate that sorting specificity is reconstituted.

### NGF Affected the Amount of its Receptors in Rafts

**Figure 6:**
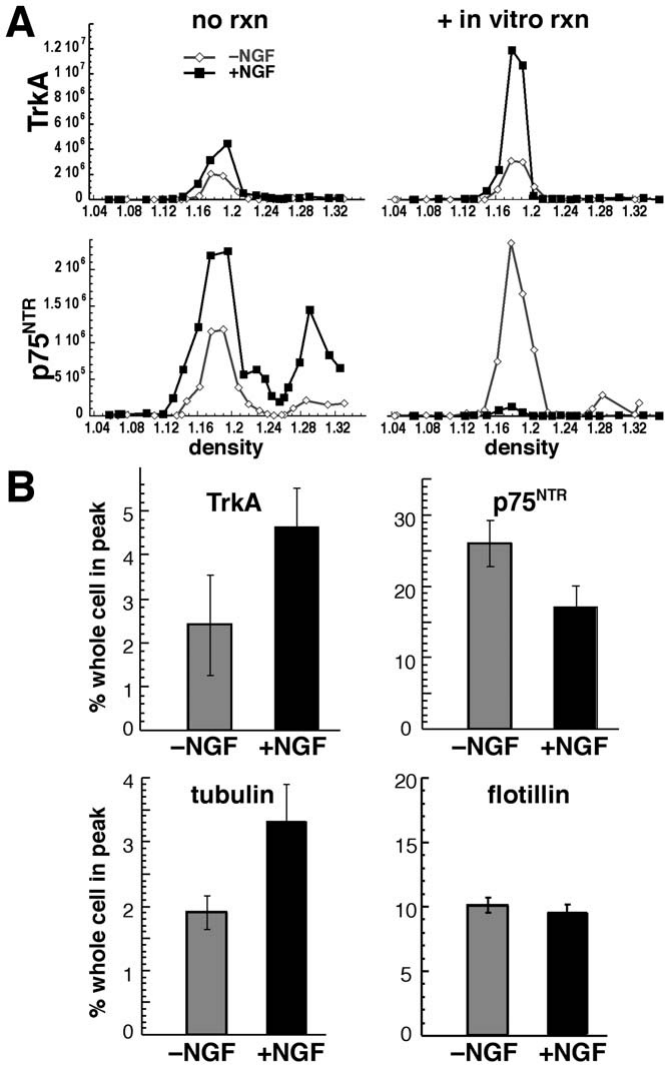
NGF differently affects association of TrkA and p75^NTR^with floating DRMs. A) Floating DRMs were isolated using Benzonase treatment after 10 min without (open diamonds) and with (closed squares) NGF treatment without (no rxn, left) and with (+in vitro rxn, right) subsequent in vitro reactions with ATP. TrkA (upper panels) and p75^NTR^ (lower panels) were quantified from western blots probed simultaneously in the same antibody solution, exposed for the same amount of time, for all conditions. Data are plotted using the same y-axis (chemiluminescence for TrkA and p75^NTR^) for all conditions. In vitro reactions had little influence on the amounts of TrkA in floating DRMs in the absence of NGF, but increased TrkA in DRMs in the presence of NGF. In vitro reactions caused p75^NTR^ to increase in the floating peak in the absence of NGF, but decrease in the presence of NGF. B) Amounts of TrkA, p75^NTR^, tubulin, and flotillin in the floating DRM peak prepared using sonication as in Figure 2, plotted as the percent of the whole cell for control (–NGF) and NGF-treated (+NGF) for cells subjected to in vitro reactions. Under these conditions, NGF caused an increase in TrkA (p<0.1) and tubulin (p<0.05) in floating DRMs, yet caused a significant decrease (p<0.05) in p75^NTR^ in floating DRMs.

We compared the effects of NGF on TrkA and p75^NTR^ in floating DRMs. Without *in vitro* reactions, NGF caused a 1.5- to 2-fold increase of both TrkA and p75^NTR^ in the floating peak (Figure 6A, no rxn). In contrast, after *in vitro* reactions, NGF caused TrkA to increase, and p75^NTR^ to decrease in the floating DRMs (Figure 6A, +*in vitro* reaction). It has been noted previously that NGF signaling enhances tubulin polymerization [45], [46]. These data, together with that showing that microtubules assemble and associate with floating DRMs during *in vitro* reactions, suggest that NGF signaling may enhance microtubule association with DRMs during these reactions. Indeed, NGF significantly increased amounts of microtubules in floating DRMs, which correlated with increased TrkA in this fraction (Figure 6B, TrkA and tubulin). In contrast, p75^NTR^ was significantly reduced in floating DRMs after NGF treatment under these conditions (Figure 6B, p75^NTR^), and flotillin was unchanged by NGF (Figure 6B, flotillin). These results indicate that NGF differently affected localization of the two co-receptors in floating DRMs under conditions where microtubules assemble and associate with membranes.

### TrkA in Detergent Resistant Endosomal Fractions

**Figure 7:**
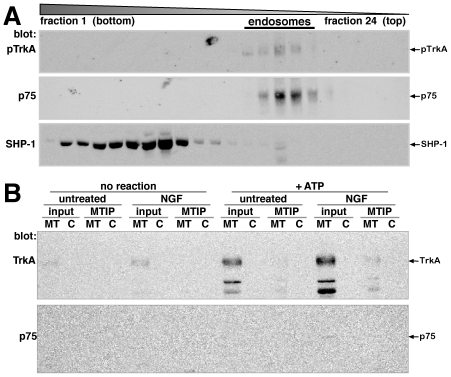
TrkA and p75^NTR^in endosomes and endosomal DRMs. A) Endosomes from cells treated 10 min with NGF. Organelles that emerged from cells mechanically permeabilized by a single pass through a Balch homogenizer were subjected to velocity sedimentation followed by equilibrium flotation as previously described [7]. Shown is the flotation equilibrium gradient from velocity gradient fraction 3, which contains TrkA and p75^NTR^ endosomes (indicated) that floated to their equilibrium density. Blots were probed with anti-pTrkA, -p75^NTR^, and -SHP-1 (indicated). B) The detergent-resistant fraction containing endosomes from untreated or NGF-treated cells before (no reaction) or after in vitro reactions (+ATP) was fractionated on iodixanol velocity gradients as previously described [34]. Pools from the bottom of the gradient containing microtubules (MT) and control samples from the top of the gradient (C) were collected for immunoprecipitations with anti-tubulin (MTIP). One-ninth of each sample was TCA precipitated before immunoprecipitation (input). Western blots were probed with anti-TrkA and anti-p75^NTR^ (indicated). pTrkA was not detected in endosomal DRMs (not shown).

One possible outcome of sorting in rafts could be for conveying receptors into different endosomes [5], [6], [7]. We asked whether TrkA could be detected in lipid rafts associated with microtubules in endosomes. We examined endosomes using organelle fractionation methods described previously [7], [34]. Organelles that emerged from mechanically permeabilized cells were subjected to velocity sedimentation followed by floatation equilibrium centrifugation on iodixanol gradients. Endosomes containing activated TrkA and p75^NTR^ were recovered from cells treated with NGF as previously described (Figure 7A) [7]. To isolate lipid rafts associated with endosomes, organelles released from mechanically permeabilized cells were treated with detergent and centrifuged at 100,000× g. The pellet was resuspended and applied to iodixanol velocity gradients that separate microtubules from other material as previously described [34]. TrkA was present in detergent-resistant endosomal fractions that contained microtubules, and amounts increased after NGF treatment and *in vitro* reactions that enhanced microtubule polymerization (Figure 7B, input, MT). When microtubules were immunoprecipitated from this fraction, TrkA was bound to them after NGF treatment and *in vitro* reactions (Figure 7B, +ATP, NGF, MTIP). In contrast, p75^NTR^ was barely detected in this detergent-resistant endosomal fraction, and none was bound to microtubules (Figure 7B, p75). Phosphorylated TrkA was not detected in the detergent-resistant endosome fraction, though it was present in endosomes (Figure 7A), which is consistent with it being dephosphorylated in DRMs extracted from whole cells (Figure 4). Since TrkA was phosphorylated in endosomes, we asked if the tyrosine phosphatase, SHP-1 was present. SHP-1 was detected only in trace amounts, or not at all, in endosomes; it was found in fractions at the bottom of equilibrium gradients, indicating that it was weakly associated and transiently bound to organelles during the first velocity gradient sedimentation (Figure 7A). The data suggest that a portion of TrkA is sorted into DRMs, dephosphorylated, and endocytosed by a mechanism that involves microtubules. p75^NTR^ does not employ this mechanism, though it associates with DRMs. Activated TrkA is endocytosed by a different mechanism that excludes the phosphatase, SHP-1 to form signaling endosomes (Figure 7A) [7].

## Discussion

In light of the effects of GM1 shown here, and many previous studies, we assume that the floating, detergent-resistant membranes derive from sphingolipid-cholesterol rafts [13], [17], so for the purpose of this discussion, we will use the term lipid rafts to refer to these membranes. The two NGF receptors, p75^NTR^ and TrkA, differed in their association with microtubules in lipid rafts in their initial response to NGF. NGF stimulates association of TrkA and NGF to newly-polymerized microtubules with lipid rafts, but not p75^NTR^. We believe that *in vitro* reactions reconstitute a sorting step that is difficult to discern in whole cells, which directly sorts TrkA and p75^NTR^ away from each other. The interaction of TrkA with microtubules in lipid rafts has implications for signal transduction, membrane traffic sorting, and axon growth.

A surprising finding is that TrkA was not phosphorylated in lipid rafts under the conditions that we have used to define them. NGF activates TrkA and rapidly stimulates its endocytosis [7], [35], [36], yet we reproducibly could not detect phosphorylated TrkA in floating DRMs under any conditions in this study, either before (Figure 4) or after *in vitro* reactions (not shown). This was the case under conditions when pTrkA was detected in detergent-sensitive (P1M) fractions and endosome fractions not treated with detergent in the same or similar experiments (Figures 4, 7) [7]. This was puzzling because co-distribution of NGF and TrkA under different conditions (*in vitro* reactions, Figure 2; GM1 treatment, Figure 3) indicate that NGF was bound to TrkA in floating DRMs, and NGF stimulated TrkA’s association with DRMs (Figure 6). The data suggest that a phospatase acts on TrkA in DRMs. Consistent with this, we detected robust amounts of the tyrosine phosphatase, SHP-1 (PTPN6) in floating DRMs (Figure 4). These results are not consistent with some previous studies that define lipid rafts using different methods [47]–[50]. Rafts defined as membranes resistant to carbonate extraction contain activated TrkA, bound to NGF and phosphorylated SHC and PLCγ [50]. Importantly, floating membranes isolated after detergent extraction and carbonate extraction have both been called lipid rafts, but the lipid and protein composition is very different for membranes isolated by these methods [13], [17]. 40% of the total TrkA was found in rafts isolated after carbonate extraction [50], compared with 2–6% in floating DRMs isolated after the detergent extraction method used here (Figures 2,3,4). In contrast, no TrkA was detected in rafts defined as Brij-58-insoluble floating membranes from mouse cerebellar and hippocampal neurons [51]. p75^NTR^ is sorts into these rafts in response to NGF and this association is blocked by a PKA inhibitor. These different results may possibly be explained by association of Trks and p75^NTR^ with other proteins in different cells, but it is more likely that different detergents define rafts with different compositions [13].

Or data is consistent with a number of other studies that distinguish RCE as a mechanism for rapid receptor signal attenuation and not for formation of persistent signaling endosomes. NGF stimulation caused a fraction of TrkA to rapidly associate with microtubules in lipid rafts (Figures 5, 6), and this association was retained in a fraction of endosomes (Figure 7B). This sorting step is probably mediated by interactions with other proteins. For example, the docking/adaptor protein, Frs3 predominantly partitions to detergent-insoluble lipid rafts, and was shown recently to bind both TrkA and microtubules and [52], [53]. Association of TrkA with lipid rafts may affect the extent to which CME or RCE play a role in TrkA endocytosis. RCE does not require caveolin expression [19], [22]. PC12 cells express little or no caveolin, and overexpression of caveolin causes rapid downregulation and diminishes the duration of TrkA signaling in response to NGF [54]. For the epidermal growth factor (EGF) receptor, ligand concentration affects sorting between CME and RCE. At low EGF concentrations, the receptor is predominantly internalized by CME, whereas at high concentrations, a greater fraction is internalized by RCE, resulting in rapid transport on microtubules to late endosomes and lysosomes for degradation [55]–[57]. Along these lines, Lakadamyali et al. [58] distinguished EGF-containing endosomes in two populations, one was dynamic and rapidly matured and transported on microtubules to join the degradative pathway, the other static and longer-lived, remaining near the cell periphery. These mechanisms may be employed by other receptors. It has been shown that disruption of microtubules and actin filaments abrogates the association of G-protein coupled receptors (β-adrenergic receptors) and Gα_s_ with lipid rafts [59]. The transforming growth factor- β (TGF-β) receptor is another example of a plasma membrane receptor that is sorted into either CME- or RCE-derived endosomes [60]. Similar to the EGFR, the receptor’s choice between CME vs. RCE dictates the rate of receptor down-regulation, the duration of signaling, and the type of Smad effectors that are activated by receptors. Our data suggest that a fraction of TrkA, like EGFR, is sorted to the RCE pathway and internalized by a microtubule-dependent mechanism. Expression of caveolin likely enhances this mechanism [54]. That TrkA was dephosphorylated in rafts suggests that RCE does not form signaling endosomes.

Hibbert et al. [25], showed that BDNF is internalized by sympathetic neurons very slowly through binding to p75^NTR^ but not TrkA, that a greater fraction of p75^NTR^ is associated with lipid rafts than TrkA, and that some NGF is associated with rafts. These data are all consistent with in PC12 cells. This paper also shows that in the absence of Trk activation, the BDNF- p75^NTR^ complex is associated with lipid rafts, but when TrkB is expressed, the amount of BDNF in rafts is reduced [25]. We found that NGF by itself slightly increased both TrkA and p75^NTR^ in rafts (Figure 6A, left plots), but after *in vitro* reactions that promote microtubule polymerization, p75^NTR^ was sorted away from rafts, while NGF and TrkA were sorted into rafts (Figure 6A, right plots and Figure 6B). Aside from possible sorting differences between TrkA and TrkB, different amounts of ligand and receptor expression, or different experimental systems and protocols, the two experiments are in fact consistent with one another if we hypothesize that in Hibbert, et al., BDNF is bound mostly to p75^NTR^ in rafts, whereas in ours, NGF was mostly bound to TrkA in rafts. Consistent with our results with TrkA in PC12 cells, TrkB is recruited into lipid rafts in cortical neurons treated with BDNF [61]. Cortical neurons do not express (or express very little) p75^NTR^. When these cells are made to express p75^NTR^, TrkB is reduced in lipid rafts [61]. Disruption of rafts by cholesterol depletion affects short-term synaptic modulation but not neuronal survival in this system, indicating that raft-borne receptors initiate local but not retrograde signaling [61]. This is consistent with our finding that TrkA was dephosphorylated in rafts, including endosomal rafts (Figures 4, 7), and supports the hypothesis that TrkA in rafts plays a local role by attracting microtubules.

Sorting of receptors into specialized signaling endosomes in neural cells may involve mechanisms that differ from those in canonical recycling and degradative endocytic pathways [28], [60], [62]. Recently, Harrington et al. [63] showed that formation of signaling endosomes containing TrkA involves a mechanism that affects actin dynamics, and that NGF, but not NT3, could activate TrkA to form persistent, retrogradely transported signaling endosomes. These results further distinguish local vs. persistent signals, and signaling endosome formation from RCE, which involves microtubules at initial stages of endocytosis [58]. It should be noted that maturation and retrograde transport of signaling endosomes and multivesicular bodies involves microtubules at later stages [64], [65].

The addition of ubiquitin (mono-ubiquitination, as opposed to poly-ubiquitination, which targets cytoplasmic proteins to the proteosome) to proteins involved in endocytosis, and to receptor tyrosine kinases themselves affects sorting between CME vs. RCE [55]–[57], [60], [62], [66]–[68]. Several proteins that play a role in endocytic sorting mechanisms (*e.g.,* Epsin, Eps15, Hrs) contain a protein domain (U1M) that binds ubiquitin [55]. Exactly how the network of ubiquitin-U1M interactions among these proteins and clathrin dictates receptor sorting is somewhat controversial [68]. In any case, when receptors are ubiquitinated, they are sorted into the RCE pathway to rapid degradation, as in the case for the EGFR in high EGF concentrations [55]. TrkA degradation is dependent on ubiquitination [69]. p75^NTR^ and Trk-family receptors affect each other’s ubiquitination and sorting into endosomes and lipid rafts. The expression of p75^NTR^ attenuates TrkA ubiquitination in HEK 293 cells, resulting in slower internalization and downregulation of TrkA [70]. A high level of expression of p75^NTR^ is thought to be the reason for low levels of TrkA ubiquitination in PC12 cells. RNAi knockdown of p75^NTR^ in PC12 cells caused increased TrkA ubiquitination and degradation after NGF treatment [70].

Our data suggest that p75^NTR^ is not endocytosed by the same mechanisms as TrkA, which is consistent with previous work [5]–[7]. Though p75^NTR^ associated with DRMs, it did not associate with microtubule rafts, and its behavior was more like flotillin in our experiments. Endosomes containing p75^NTR^ overlapped with only a subset of flotillin organelles in size and density, however [7]. Flotillin has been shown to mediate an endocytic pathway that is distinct from both CME and RCE [71]. EGF signaling affects flotillin endocytosis and the actin cytoskeleton to cause cell spreading [72]. In our experiments, flotillin in rafts was reduced by GM1 treatment (Figure 3), but not by *in vitro* reactions that increased microtubules in rafts (Figure 2). Flotillin was not affected by NGF (Figure 6). Thus, while flotillin-dependent endocytosis may be present in PC12 cells, our data suggests that flotillin trafficking is not regulated by NGF receptor signaling, and does not employ microtubule rafts.

If raft-associated TrkA is not incorporated into signaling endosomes, its attraction of microtubules to the plasma membrane may play a different role. Microtubules have been shown to be involved in maintaining polarity in neurons and other cell types both by selective anterograde delivery of secretory vesicles and selective retrieval via endocytosis [73], [74]. An important local signal initiated by neurotrophins is to stimulate axon growth. Neurotrophins cause axon growth and are attraction cues for axon guidance, which employs some mechanisms in common with those that induce cell polarity. Lipid rafts have been shown to play a role in cytoskeleton organization and its interaction with the plasma membrane to dictate cell polarity [73], [75]. There is evidence that rafts are involved in coordinating interactions between actin filaments and microtubules. For example, integrins cause local stabilization of microtubules at the leading edge in migrating cells through a mechanism that involves Rho [76], and rafts are endocytosed in detaching cells through Rac1 and actin [77].

In addition to a role for lipid rafts in growth cone guidance [14], [15], there is evidence that interactions with microtubules and lipid rafts play a role in axon guidance [78]. NGF influences microtubule dynamics at axon tips to cause axon growth [10]. This effect is mediated by TrkA signaling through PI-3 kinase and GSK-3β to control the axon tip localization of microtubule plus-end binding protein APC [10]. NGF, through this mechanism, acts as a powerful attractant to growth cones, powerful enough to overcome the strong inhibitory influence of chondroitin sulfate proteoglycans in a nerve regeneration model [78]. Our data showing that TrkA associates with microtubules in lipid rafts, and that NGF enhanced polymerization of microtubules associated with lipid rafts, are consistent with a role for lipid rafts in axon guidance cues driven by NGF. The data suggest that NGF, through TrkA, mediates axon guidance by attracting microtubules to lipid rafts. These local signals that affect the cytoskeleton at the cell cortex do not require persistent signaling, which explains TrkA’s rapid dephosphorylation in microtubule rafts. A decrease in p75^NTR^ in microtubule-associated rafts in response to NGF and TrkA can help explain how attraction signals overcome repulsive ones initiated by p75^NTR^ and its other co-receptors, NgR, a GPI-linked protein that associates with lipid rafts [79], and Lingo-1 [11], [80]. Differential association of TrkA and p75^NTR^ with microtubules and rafts may determine the outcome of attraction vs. repulsion.

## Materials and Methods

Most general chemicals were purchased from Sigma (St. Louis, MO). NGF was a kind gift of William Mobley. Horse serum and foetal calf serum were from Life Technologies (Gaithersburg, MD). Iodixanol (Optiprep^TM^) was from Nycomed Pharma, Inc. (Oslo, Norway). ^125^I radioisotope was obtained from NEN^TM^ Life Science Products Inc. (Boston, MA). The anti-rat TrkA antibody (RTA) was a kind gift of Dr. Louis Reichardt (University of California, San Francisco), and was also purchased from Upstate Biotechnology (Lake Placid, NY). Phospho-TrkA antibody was from Cell Signalling Technologies (Danvers, MA). Anti-p75^NTR^ and anti-SHP-1 were obtained from Santa Cruz Biotechnology (Santa Cruz, CA) and Covance/BabCo (Princeton, NJ/Berkeley, CA). Anti-flotillin was purchased from Transduction Laboratories (Lexington, KY), sc11 anti-TrkA from Santa Cruz Biotechnology (Santa Cruz, CA), and anti-β-tubulin was obtained from Sigma. Anti-mouse and –rabbit-HRP was obtained from Amersham Biosciences (Buckinghamshire, UK, or Piscataway, NJ).

### Cell Treatments and In Vitro Reactions

Wild-type PC12 cells were obtained from Lloyd Greene (Columbia University) and grown on collagen-coated plates in RPMI 1640, 5% fetal calf serum, 10% horse serum as described [81]. ^125^I-NGF was prepared as previously described [36]. In some experiments biotinylated lactoperoxidase was used (Sigma) and removed by binding to neuravidin beads (Pierce, Rockfork, IL) prior to separation of radiolabeled protein from free iodine.

PC12 cells (typically 0.5–1×10^9^) were harvested in PBS and washed in cold PEE (PBS/1mM EGTA/1mM EDTA), PGB (PBS/0.1% glucose/0.1% BSA) as described [35], [36]. For comparison of treatment conditions, equal volumes of cell suspension were dispensed. Where NGF was added, 1 nm NGF or ^125^I-NGF was bound to a rotating cell suspension 1 h at 4°C in PGB. Unbound ligand was removed by a wash in PGB to avoid fluid-phase endocytosis. For GM1 treatments, cells were harvested, separated into two equal aliquots, and incubated in either serum-free media with 65 µM GM1, or serum-free media alone, for 5 hours at 37°C in a 5% CO_2_ incubator. After this incubation period the cells were washed and ^125^I-NGF was bound to the cells above.

Cells were fractionated directly or warmed in PGB to 37°C exactly 2, 10, or 30 min, followed by a temperature-quench in ice water. Cells were then centrifuged 100 × g for 3 minutes and washed with 5 ml PEE, followed by a wash with 5 ml buffer B (cytoplasmic ionic composition: 38 mM each of the K^+^ salts of aspartic acid, glutamic acid and gluconic acid, 20 mM MOPS pH 7.1 at 37°C, 10 mM potassium bicarbonate, 0.5 mM magnesium carbonate, 1 mM EDTA, 1 mM EGTA), and resuspended in buffer B with 5 mM reduced glutathione (abbreviated BB+G). Protease inhibitors were added to a final concentration of 174 µg/ml PMSF, 1 µg/ml o-phenathroline, 10 ng/ml pepstatin, 10 ng/ml chymostatin, 10 ng/ml leupeptin, and 10 ng/ml aprotinin. Cells were mechanically permeabilized by a single passage through a Balch homogenizer in buffer B as described [7], [35], [36].

Where *in vitro* reactions were performed, the permeabilized cell suspension was split and one sample was warmed for 15 minutes at 37°C with an ATP regenerating system (1 mM ATP, 8 mM creatine phosphate, 5 mg/ml [240 units/mg] creatine kinase). After reactions, the samples were quenched in ice water for 3–5 minutes.

Dorsal root ganglia neurons were obtained from 30–40 embryos at stage E13. Ganglia were dissected from embryos in Leibovitz’s L-15 media (Gibco BRL), washed Eagles Balanced Salt Solution, and treated with 0.05% trypsin for 25 min. Cells were centrifuged 200 × g 4 min, then resuspended and plated on polylysine/laminin-coated 10 cm dishes and cultured in MEM, 10% FBS, 0.2% glucose, 2 mM glutamine with antibiotics (Pen/Strep) in the presence of 1.7 nM NGF for 4–10 days. The yield was about 8 million cells total. To prepare DRMs from neurons, cells were rinsed in warm PBS, then cold PEE was added and plates placed on ice for 30 min. This caused the cells to lift from the dish without breaking apart. Cells were recovered by centrifugation bound to NGF, warmed, and DRMs were prepared as described above for PC12 cells, except that neurons were swollen in hypo-osmotic 0.1 × BB prior to mechanical permeabilization with the Balch homogenizer.

### Velocity and Equilibrium Centrifugation

Large membranes were removed from the mechanically permeabilized cell suspension by centrifugation at 1000 × g [7], [35], [36]. Gradients used iodixanol mixed with buffer B plus glutathione (BB+G) as media; continuous gradients were prepared using a two-chamber mixer. The supernatant of the 1000 × g centrifugation (S1) was layered over 0–30% velocity gradients. For two dimensional separations (velocity followed by equilibrium gradients), five velocity gradient fractions were collected, mixed with 60% iodixanol to a concentration of 32.5% or greater, and overlaid with a continuous 0–30% iodixanol/BB+G gradient and centrifuged to equilibrium (16–18 hr). Refractive indices were measured using an Abbe refractometer (Bausch and Lomb) and converted to density using the formula ρ=R.I.× 3.4319–3.5851, which was determined empirically by weighing known concentrations of iodixanol in buffer B.

### DRM Preparations

Cracked cell suspensions were centrifuged at 1,000 × g for 10 minutes (pellet=P1; supernatant=S1). The P1 pellet was resuspended in buffer B containing protease inhibitors. Triton X-100 or IGEPAL (Sigma) was added to a final concentration of 1% and the sample was vortexed and left on ice for at least 1 hour. The sample was centrifuged at 10,000xg for 10 minutes to separate the detergent-soluble membranes (supernatant=P1M) from the detergent-insoluble pellet. This pellet was then resuspended in 180 µl BB with protease inhibitors and Triton X-100 was again added to a 1% concentration. Iodixanol (OptiPrep^TM^) was added to a 40% concentration and the samples sonicated for 2 × 5-second pulses. This step was necessary as in preliminary experiments without sonication, DNA and cytoskeletal elements often clogged the needle of the gradient fraction collector. Alternatively, instead of sonication, 2 µl Benzonase (Novagen, Madison, WI) was added to the resuspended detergent resistant pellet and the sample incubated on ice for 1 hour to break up DNA prior to adding iodixanol. The method used for each set of experiments presented is identified in the figure legends.

Samples were placed into 5 ml ultracentrifuge tubes and 10–40% (for sonicated samples) or 15–48% (for Benzonase-treated samples) continuous iodixanol gradients in BB were poured over the top of the samples at 4°C. Gradients were centrifuged to equilibrium at 100,000xg for 16–18 hours. Approximately 200 µl fractions were collected from the bottom of the ultracentrifuge tube. Radioactivity in each fraction was determined using a gamma counter. The refractive index of each gradient fraction was measured and the density calculated based on a formula determined empirically by weighing iodixanol/BB standards of known concentration. TCA was added to each fraction to a final concentration of 10%, and left overnight at 4°C to precipitate protein. Protein precipitates were recovered by centrifugation at 3,500 × g for 35 minutes or 10,000× g for 20 min, then washed in ice-cold acetone and re-centrifuged. Precipitates were air dried at room temperature and 7 M urea sample buffer (7 M urea, 125 mM Tris HCl pH 6.95, 0.1% w/v bromophenol blue with 100 mM DTT) was added and samples were heated to 55°C for 15 minutes before analysis by SDS-PAGE.

### Microtubule Immunoprecipitations

For immunoprecipitation of microtubules, 10 µM taxol was added to the resuspended detergent-resistant pellet (treated with Benzonase) and gradients to stabilize microtubules. Where indicated, 25 µg/ml biotinylated tubulin and 12.5 µM taxol (both from Cytoskeleton, Inc., Denver, CO) were added during the last 5 min of 15 min *in vitro* reactions prior to preparation of DRMs. For microscopy, the sample was brought to 10% glycerol, 5% BSA and 1∶100 Texas Red-streptavidin (Vector Laboratories, Burlingame, CA) were added and incubated on ice for 2 hr. The permeabilized cells were washed 3X in buffer B with 0.1% BSA and recovered by centrifugation at 100 × g, 3 min. The sample was resuspended in buffer B with 20% glycerol, mixed with VectaShield (Vector Laboratories) and viewed with a 100x objective on a Nikon E800 with Hamamatsu ORCA II detector. The presence of floating membranes was confirmed by western blots of gradient fractions in parallel gradients on one half of the sample. Fractions containing the floating membranes of density 1.21- 1.15 g/ml, determined by refractive index measurements, were pooled and buffer components were added to 10% glycerol, 1% BSA,150 mm M NaCl, 50 mM Tris pH 7.7, 1% IGEPAL, 1 mM EDTA, and 10 µM taxol. Where indicated, samples were incubated overnight at 4°C with anti-α-tubulin (DM1A, Sigma) or anti-TrkA (RTA, Upstate Biotechnology). Antibodies were recovered with Pierce Ultralink ProteinA/G beads; biotinylated microtubules were recovered with Neuravidin beads. Bead suspensions were rotated 1 hr at 4°C, then recovered by centrifugation 1000 × g for 5 min, washed twice in wash buffer (10% glycerol, 150 mm M NaCl, 50 mM Tris pH 7.7, 1% IGEPAL, 1 mM EDTA, 10 µM taxol), then once in 0.01X wash buffer, and resuspended in SDS-PAGE sample buffer.

Microtubules were immunoprecipitated from the detergent-resistant endosome fraction exactly as described [34]. Briefly, PC12 cells were treated with or without NGF and subjected to *in vitro* reactions as above. The organelles that emerged from mechanically permeabilized cells, which have been shown to contain TrkA bound to NGF [7], [35], [36], were incubated with 1% IGEPAL. Detergent-resistant material was concentrated by centrifugation at 100,000 × g, 1 hr, resuspended and applied to iodixanol velocity gradients. Fractions at the bottom of gradients containing microtubules, and control samples from the top of the gradient, were individually pooled and immunoprecipitated with anti-α-tubulin as described above except without taxol.

### SDS-PAGE and Western Blotting

SDS-PAGE gels were run and western blotted to nylon-reinforced nitrocellulose (Schleicher and Schull, Dassel, Germany) as described [82]. Where different treatment conditions are compared in one experiment, all blots were incubated in the same antibody solution on the same day and exposed for the same amount of time. Blot incubations were performed in 5% nonfat dry milk, 150 mM NaCl, 50 mM Tris pH 7.7, 0.05% Tween 20, or conditions specified by the antibodies’ manufacturer. Secondary anti-mouse or –rabbit antibodies coupled to HRP (Amersham) were used and chemiluminescent signals generated by Amersham ECL^TM^ or Super Signal West Pico (Pierce). The blot was either exposed to X-ray film (Fuji medical x-ray film, HR-G 30) or exposed directly in a Fujifilm Intelligent Dark Box II with a cooled CCD camera (LAS-1000, or LAS-3000, Fuji Photo Film Co. Ltd, Japan). Blots were stripped of antibodies for re-probing with Restore (Pierce), TBS pH 2.0, or in 0.5 M NaCl, 0.2 M glycine, pH 2.8.

### Image Analysis and Calculations

Chemiluminescent data captured directly or on film by the LAS-1000 or 3000 image analyser were analysed using Fuji Image Gauge software (Fuji Film Co. Ltd). For each protein these area values were added together to give a ‘total gradient' or ‘total DRM' protein value. The protein band intensities for the S1 and P1M samples were calculated by taking into account the volume that was loaded onto the gel compared with the original S1 and P1M sample size. Amounts of ^125^I-NGF in each fraction were determined using a gamma counter. For each protein (or ^125^I-NGF), the amount in the floating DRM peak was calculated and expressed as a percentage of that particular protein in all cell fractions. P values were calculated using the students t-test.
